# An adult B-cell precursor acute lymphoblastic leukemia with multiple secondary cytogenetic aberrations

**DOI:** 10.1186/s13039-014-0060-0

**Published:** 2014-09-10

**Authors:** Walid AL-Achkar, Abdulsamad Wafa, Moneeb Abdullah Kassem Othman, Faten Moassass, Abdulmunim Aljapawe, Thomas Liehr

**Affiliations:** Department of Molecular Biology and Biotechnology, Human Genetics Division, Atomic Energy Commission of Syria, P.O. Box 6091, Damascus, Syria; Department of Molecular Biology and Biotechnology, Mammalians Biology Division, Atomic Energy Commission, Damascus, Syria; Institute of Human Genetics, Jena University Hospital, Jena, Germany

**Keywords:** Acute lymphoblastic leukemia, Secondary chromosomal abnormalities, Philadelphia chromosome, Fluorescence in situ hybridization, Array-proven multicolor banding, Prognostic factors

## Abstract

**Background:**

We report a clinically diagnosed acute lymphoblastic leukemia (ALL) with yet unreported secondary chromosomal aberrations.

**Results:**

A complete cytogenetic and molecular cytogenetic analysis, using GTG banding, fluorescence in situ hybridization (FISH) and array-proven multicolor banding (aMCB), for a female patient with clinically diagnosed ALL and immunophenotypically confirmed pre-B ALL (FAB classifications), revealed the presence of a complex structural rearrangement, der (2) (20qter- > 20q13.33::2q21- > 2p14::2q21 > 2qter) along with t (9;22) (q34;q11), t (12;14) (q12;p12) and a monosomy of chromosome 7.

**Conclusions:**

Molecular cytogenetic studies are suited best for identification and characterization of chromosomal rearrangements in acute leukemia. Single case reports as well as large scale studies are necessary to provide further insights in karyotypic changes taking place in human malignancies.

## Background

Acute lymphoblastic leukemia (ALL) is a heterogeneous disease characterized by multiple subtypes [1]. To date, several structural and numerical chromosomal abnormalities have been characterized in ALL and according to the WHO classification the following, seven genetic subtypes are defined for B lymphoblastic leukemia, t (9:22) (q34;q11.2), 11q23 traslocations, t (12;21) (p13;q22), t (1;19) (q23;p13.3), t (5;14) (q31;q32), hyperdiploidy and hypodiploidy [2]. Among the genetic subtypes, Philadelphia (Ph) chromosome, which results from a reciprocal translocation between Abelson (*ABL1*) from chromosome 9 and breakpoint cluster region (*BCR*) from chromosome 22, is the most frequent cytogenetic aberration which is found in ~ 25% of adult ALL cases, and in more than 50% of patients, aged 50 years or more [3,4]. The presence of the BCR-ABL1 rearrangement worsens the prognosis of ALL and represents the most significant adverse prognostic marker that influences the disease outcome [5]. Ph positive (Ph+) ALL is a more aggressive disease than chronic myeloid leukemia (CML), indicating that other factors than BCR-ABL1 are involved in its development and progression [5,6]. Ph + precursor-B-ALL is highly aggressive, frequently resistant to chemotherapy and with a short survival time [6,7]. Here, we are presenting a Ph + pre-B-ALL case with yet unreported translocation events involving six different chromosomes and a monosomy 7. These chromosomal rearrangements appeared after unsuccessful chemotherapy treatment.

## Case presentation

A 31-year-old woman was diagnosed as suffering from ALL in September 2011. Anemia, thrombocytopenia, diarrhea, fatigue and weight loss were the indicative symptoms. She was treated as follows: after the first GM-ALL protocol (phase I and II) failed, Flag-IDA protocol was used, which also did not succeed. Then again GM-ALL protocol (phase I and II) was applied and after being unsuccessful hyper-CVAD was applied. At this point the first cytogenetics and hematology were determined. The patient's hematologic parameters were white blood cells (WBC) at 123×10^9^/l, consisting of 12% neutrophils, 75% lymphocytes, 11% monocytes and 1% basophiles. Red blood cell (RBC) count was 3.26×10^6^/mm^3^, hemoglobin level 9.7 g/dl and the platelet count 34×10^9^/l. Serum lactate dehydrogenase (LDH) value was 2,712 U/l (normal value up to 480 U/l), serum alkaline phosphates value 208 U/l (normal value up to 128 U/l), serum alanine aminotransferase 198 U/l (normal value up to 40 U/l) and serum aspartate aminotransferase value 139 U/l (normal value up to 40 U/l). The patient was treated further according to standard ALL chemotherapy protocols for fourteen months, however, without clinical success of chemotherapy. Unfortunately she died under the treatment.

## Results

**Figure 1:**
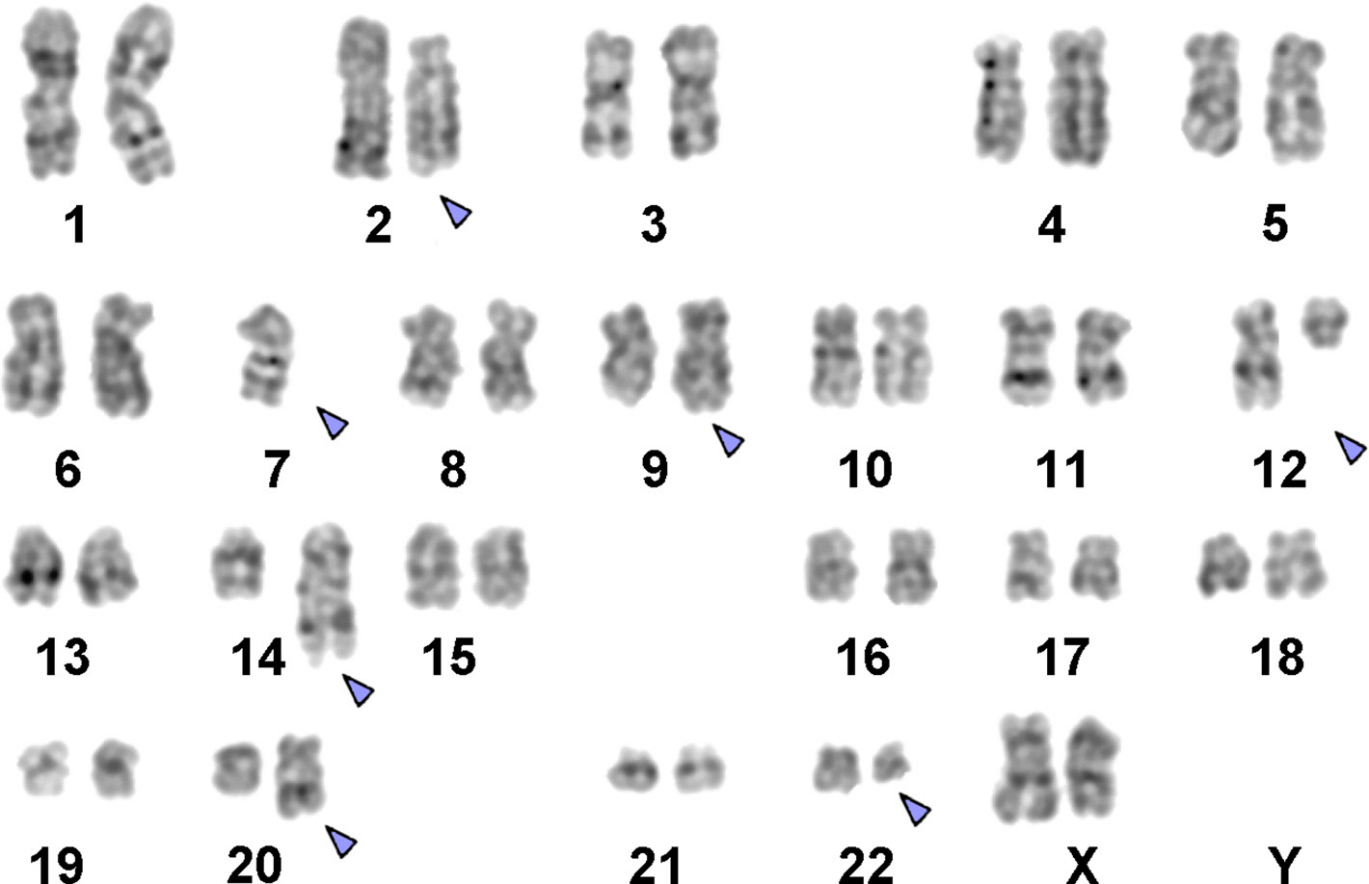
**GTG-banding revealed a 45, XX-7, der (2) t (2;20), t (9;22), t (12;14).** All derivative chromosomes are shown with arrows.

**Figure 2:**
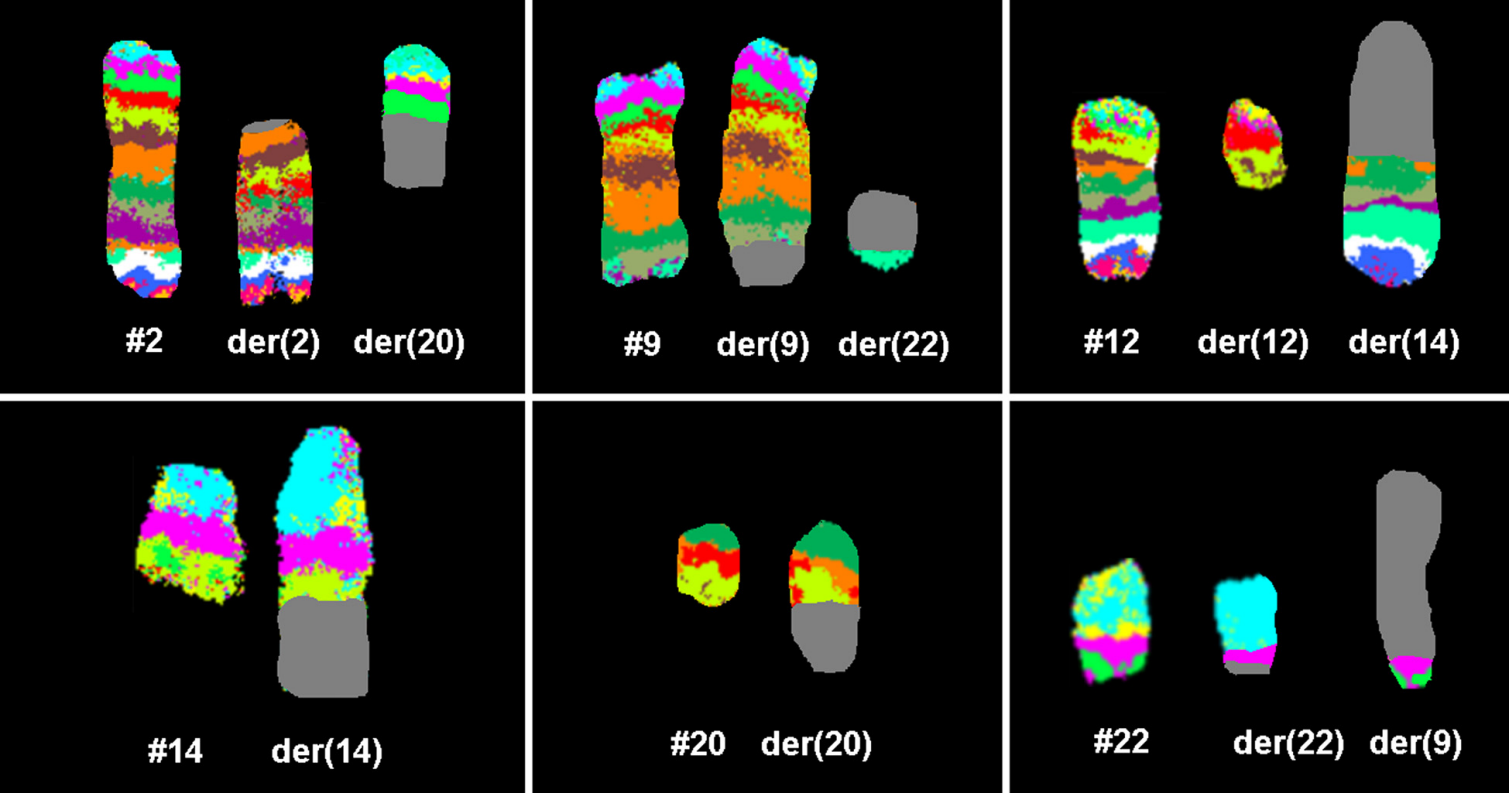
**Array-proven multicolor banding (aMCB) was applied to characterize the breakpoint locations.** Each image shows the results of MCB analysis using probe sets for chromosomes 2, 9, 12, 14, 20 and 22. The normal chromosomes are shown in the left side of each image and the derivative chromosomes on the right. The MCB-probes unstained regions on the derivative chromosomes are shown in gray. Abbreviations: # = chromosome; der = derivative chromosome; Ph = Philadelphia chromosome.

A sample of a female patient diagnosed as pre B-ALL, according to FAB classifications, was received after the completion of three different protocols of chemotherapy. The conventional cytogenetics analysis by GTG banding revealed the karyotype as 45, XX, -7, der (2) t (2;20) (?;?), t (9;22) (q34;q11), t (12;14) (q?;p?) [12] / 46, XX, t (12;14) (q?;p?) [10] (Figure 1). The dual color FISH using the probe specific for BCR and ABL and WCP probes specific for chromosomes 2, 7, 12, 14 and 20 confirmed the presence of BCR/ABL fusion on der (22) (data not shown), and the presence of the other rearrangements. To further characterize the breakpoints, aMCB was performed, as previously reported [8] (Figure 2) and the final karyotype was redefined as: 45, XX,-7, der (2) (20qter- > 20q13.33::2q21- > 2p14::2q21 > 2qter), t (9;22) (q34;q11), t (12;14) (q12;p12) [12] / 46, XX, t (12;14) (q12;p12) [10].

The abnormal cell population showed the following immunophenotype, which was consistent with pre-B-ALL (FAB classifications): CD45+, HLADr+, CD117+, CD34+, CD19+, CD10+, CD38+ and expressed CD123 and CD11c (52%) heterogeneously. The abnormal cells negatively reacted with antibodies to CD5, CD64 and CD3.

## Conclusions

We characterized a Ph + adult pre-B-ALL case with a complex secondary chromosomal abnormality, a translocation and a monosomy 7. According to the literature, not a single case of ALL showed a der (2) (20qter- > 20q13.33::2q21- > 2p14::2q21- > 2qter) plus a t (12;14) (q12;p12) [9]. Moreover, a t (12;14) (q12;p12) was observed only in two cases of mantle cell lymphoma [9] and in a case of acute myeloid leukemia [10]. On the other hand, the chromosomal bands, 2p14, 2q21, 12q12, and 14p12 are listed in 5, 32, 20, and 4 cases, respectively, in other rearrangements involving different chromosomes than the ones which are involved in the present case, in previously reported ALL cases [9]. In addition, inv (2) with 2q21 as one of the breakpoints has also been reported in 3 cases of ALL [9].

Till date, several chromosomal aberrations such as t (9;22), t (4;11), t (1;9), and hyperdiploid or hypodiploid karyotype have been associated with the prognostic outcome in ALL cases. Apart from t (9;22) (q34;q11)/BCR-ABL and t (4;11) (q21;q23)/MLL-AF4, an elevated white blood cell count, age over 40 and non-responders/slow responders to chemotherapy are commonly regarded as high risk criteria in ALL [11]. Monosomy 7, as a sole secondary abnormality, is also related with a poor prognosis and shorter survival in adult ALL cases [12,13]. In addition, deletions of 7p confer with an inferior outcome in children with ALL, regardless of the presence of other poor prognostic features, whereas deletions of 7q are not associated with an adverse outcome [14]. The tendency for an adverse prognosis in patients with secondary loss of chromosome 7 or 7p in Ph + ALL may be the cumulative result of these events. Mullighan et al. [15] recently described a deletion of IKZF1 gene which encodes the transcription factor Ikaros, located on 7p12 in 83.7% of Ph + ALL cases but not in chronic-phase CML, suggesting that loss of Ikaros, a prototypical member of the Krüppel-like zinc finger (ZnF) transcription factor subfamily, which is required for normal hematopoietic differentiation and proliferation, particularly in lymphoid lineages, [16–18] is an important step in the progression of Ph + ALL. Recently, two of seven myeloproliferative neoplasms patients with loss of IKZF1 due to monosomy 7 have also been reported which suggests that IKZF1 may represent an important tumor-suppressor gene affected by monosomy 7 [19].

The presence of the underlying BCR/ABL gene rearrangement in CD10 B-cell precursor ALL has been reported previously [20] and it has already been demonstrated that the occurrence of BCR-ABL positive ALL in comparison to BCR-ABL negative disease represents a subgroup with a worse prognosis within the CD10+ B-lineage ALL [21].

In conclusion, the present case is a de novo case of adult pre-B-ALL with yet unreported translocation events involving six different chromosomes in addition to monosomy 7.

## Materials and methods

### Chromosome analysis

Chromosome analysis using GTG-banding was performed according to standard procedures [22] 12 months after ignition of the chemotherapeutic treatment. A minimum of 20 metaphase cells derived from unstimulated bone marrow culture were analyzed. Karyotypes were described according to the International System for Human Cytogenetic Nomenclature [23].

### Molecular cytogenetics

Fluorescence in situ hybridization (FISH) using LSI BCR/ABL three-color dual-fusion translocation probe (Abbott Molecular/Vysis, Des Plaines, IL, USA) was applied according to manufacturer's instructions together with a whole chromosome painting (WCP) probe for chromosomes 2, 7, 12, 14 and 20 (MetaSystems, Altlussheim, Germany) [22]. FISH using the corresponding chromosome specific array-proven multicolor banding (aMCB) probe sets based on microdissection derived region-specific libraries was performed as previously reported [8]. A minimum of 20 metaphase spreads were analyzed, using a fluorescence microscope (AxioImager.Z1 mot, Carl Zeiss Ltd., Hertfordshir, UK) equipped with appropriate filter sets to discriminate between a maximum of five fluorochromes plus the counterstain DAPI (4',6- diamino-2-phenylindole). Image capture and processing were performed using an ISIS imaging system (MetaSystems).

### Flow cytometric immunophenotype

Flow cytometric analysis was performed using a general panel of fluorescent antibodies against the following antigens typical for different cell lineages and cell types: CD1a, CD2, CD3, CD4, CD5, CD8, CD10, CD11b, CD11c, CD13, CD14, CD15, CD16, CD19, CD20, CD22, CD23, CD32, CD33, CD34, CD38, CD41a, CD45, CD56, CD57, CD64, CD103, CD117, CD123, CD138, CD209, CD235a and CD243; In addition to antibodies to Kappa and Lambda light Chains, IgD, sIgM, and HLADr. All antibodies were purchased from BD Biosciences. Samples were analyzed on a BD FACSCalibur™ flow cytometer. Autofluorescence, viability, and isotype controls were included. Flow cytometric data acquisition and analysis were conducted by BD Cellquest™ Pro software.

## Consent

Written informed consent was obtained from the patient for publication of this Case Report. A copy of the written consent is available for review by the Editor-in-Chief of this journal.
